# Rodent models of tumours of the central nervous system

**DOI:** 10.1002/1878-0261.13729

**Published:** 2024-09-26

**Authors:** Sebastian Brandner

**Affiliations:** ^1^ Department of Neurodegenerative Disease UCL Queen Square Institute of Neurology and Division of Neuropathology, The National Hospital for Neurology and Neurosurgery, University College London Hospitals, NHS Foundation Trust London UK

**Keywords:** brain tumour, Cre‐lox system, CRISPR Cas9 system, genetically modified mouse model, glioblastoma, medulloblastoma

## Abstract

Modelling of human diseases is an essential component of biomedical research, to understand their pathogenesis and ultimately, develop therapeutic approaches. Here, we will describe models of tumours of the central nervous system, with focus on intrinsic CNS tumours. Model systems for brain tumours were established as early as the 1920s, using chemical carcinogenesis, and a systematic analysis of different carcinogens, with a more refined histological analysis followed in the 1950s and 1960s. Alternative approaches at the time used retroviral carcinogenesis, allowing a more topical, organ‐centred delivery. Most of the neoplasms arising from this approach were high‐grade gliomas. Whilst these experimental approaches did not directly demonstrate a cell of origin, the localisation and growth pattern of the tumours already pointed to an origin in the neurogenic zones of the brain. In the 1980s, expression of oncogenes in transgenic models allowed a more targeted approach by expressing the transgene under tissue‐specific promoters, whilst the constitutive inactivation of tumour suppressor genes (‘knock out’)‐often resulted in embryonic lethality. This limitation was elegantly solved by engineering the Cre‐lox system, allowing for a promoter‐specific, and often also time‐controlled gene inactivation. More recently, the use of the CRISPR Cas9 technology has significantly increased experimental flexibility of gene expression or gene inactivation and thus added increased value of rodent models for the study of pathogenesis and establishing preclinical models.

## Graphical abstract

We describe the evolution of experimental systems, illustrated here in a timeline to show changing popularity over time. Starting with chemical and viral carcinogenesis, systems then developed into increasingly sophisticated genetically modified animal models. The literature cited in this article corresponds to the arrows in this illustration and the schematics illustrate the mechanism of action described in this article.

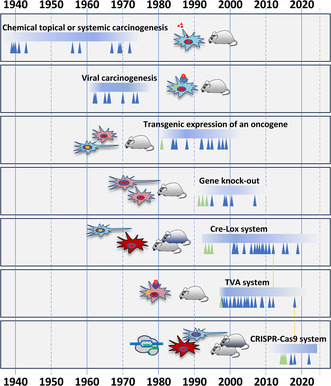

**Abbreviations**
*Akt*
: A designation for protein kinase B, encoded by the genes AKT1, AKT2, and AKT3
APC: Adenomatosis polyposis coli, a tumour suppressor gene/protein
ARF: alternate reading frame
ATRX: Alpha Thalassemia/Mental Retardation Syndrome X‐Linked, chromatin remodeller
BRAF: v‐Raf murine sarcoma viral oncogene homologue B, a serine–threonine protein kinase
CDK 4: Cyclin‐dependent kinase 4, member of the Ser/Thr protein kinase family
CNS: central nervous system, anatomical system comprising brain and spinal cord
Cre ER(T): Cre‐recombinase is activated through a tamoxifen inducible system, i.e. an Oestrogen receptor is transgenically expressed (Oestrogen Receptor (tamoxifen))
Cre‐lox: Cre protein (encoded by the locus originally named as Cause of recombination; lox (or lox P) Locus of X [crossing]‐over P1) is a site on the bacteriophage P1 consisting of 34 bp
CRISPR Cas9: Clustered regularly interspaced short palindromic repeats, a gene editing technology; Cas9: CRISPR associated protein 9
EGFR: Epidermal growth factor receptor, transmembrane protein that is a receptor for members of the epidermal growth factor family (EGF family) of extracellular protein ligands
FGF: Fibroblast growth factor, a cell signalling protein
G TVA: tv‐a receptor under the control of GFAP
GFAP: Glial fibrillary acidic protein, a structural protein expressed in astrocytes and in a range of other cells in the developing and adult CNS
GFP: Green fluorescent protein, a protein that exhibits green fluorescence when exposed to light in the blue to ultraviolet range, originally derived from a jellyfish, but widely modified for cell and tissue labelling purposes
GLI: Glioma‐Associated Oncogene Family (Zinc Finger protein) protein in the hedgehog signalling pathway
HSP: heat shock protein (chaperone proteins)
IDH: Isocitrate dehydrogenase, a gene involved in the Krebs cycle, and mutations in the IDH1 or less commonly, IDH2 gene are found in the CNS tumours astrocytoma and oligodendroglioma
IGFBP: Insulin‐like growth factor binding protein
*Ink4a/Arf*
: inhibitors of CDK4, member of a family of cyclin
K‐ras: Kirsten rat sarcoma virus, named after Kirsten, a pathologist
MAP kinase: mitogen‐activated protein kinase
MMEJ: microhomology‐mediated end joining, a DNA repair mechanism
MYC: Myelocytomatosis (v‐MYC avian virus, c‐MYC – cellular), a proto‐oncogene
N TVA: tv‐a receptor under the control of nestin
NF1, NF2: neurofibromatosis
NHEJ: non‐homologous end joining, a DNA repair mechanism
OPC: Oligodendrocyte precursor cells
PDGFB: Platelet‐derived growth factor beta
PTCH: Patched, a receptor involved in cell signalling (sonic hedgehog pathway)
PTEN: Phosphatase and tensin homologue, a tumour suppressor gene/protein
RB: Retinoblastoma, a tumour suppressor gene/protein
RCAS: Replication‐Competent Avian Sarcoma‐Leukosis Virus Long Terminal Repeat with a Splice Acceptor
SHH: Sonic hedgehog, a ligand involved in cell signalling
SMO: Smoothened, a protein of the sonic hedgehog pathway
SUFU: Suppressor of fused homologue, negative regulator of hedgehog signalling
SVZ: subventricular zone (the area adjacent to the lateral ventricles in the brain)
Tva: Tumour virus (tv)‐A receptor; the receptor for avian leucosis virus‐subgroup A, ALV‐A
WT: Wild‐type

## 1 Introduction

This review provides an overview of the development of model systems of carcinogenesis in the central nervous system. Starting from the very beginnings of experimental induction of CNS tumours through chemical and viral oncogenesis, the review follows the historical timeline of the beginnings of transgenesis, and its subsequent stepwise refinements. It shows the long pathway of technological advancements in this field, and importantly, how developments of disease models interacted with corresponding discoveries of disease mechanisms in human CNS tumours. Without the discovery of driver mutations in human tumours, none of the mouse models would have been able model CNS tumours beyond phenotypic similarities. On the other hand, many of the genetic events found in human tumours could not have been understood without systematic mechanistic analysis in mouse models. It also highlights the importance of a wide diversity of model systems, for gene discovery and preclinical testing. Early models primarily aimed at the recapitulation of tumours that have similarities to the histological appearances of human counterparts, regardless of a correspondence between human disease and the model system simply because the histology of a tumour was the only available characteristic. The discovery of genetic lesions underpinning human tumours required adaptation of model systems, as well as the development of technologies (such as gene knockout, gene knockin, conditional knockout, etc.). Once high‐throughput technologies allowed a more comprehensive screening of human cancers, it also led to ‘upgrading’ and ‘upscaling’ of model systems, now providing much greater versatility, example using CRISPR Cas9 or base and prime editing.

**Fig. 1:**
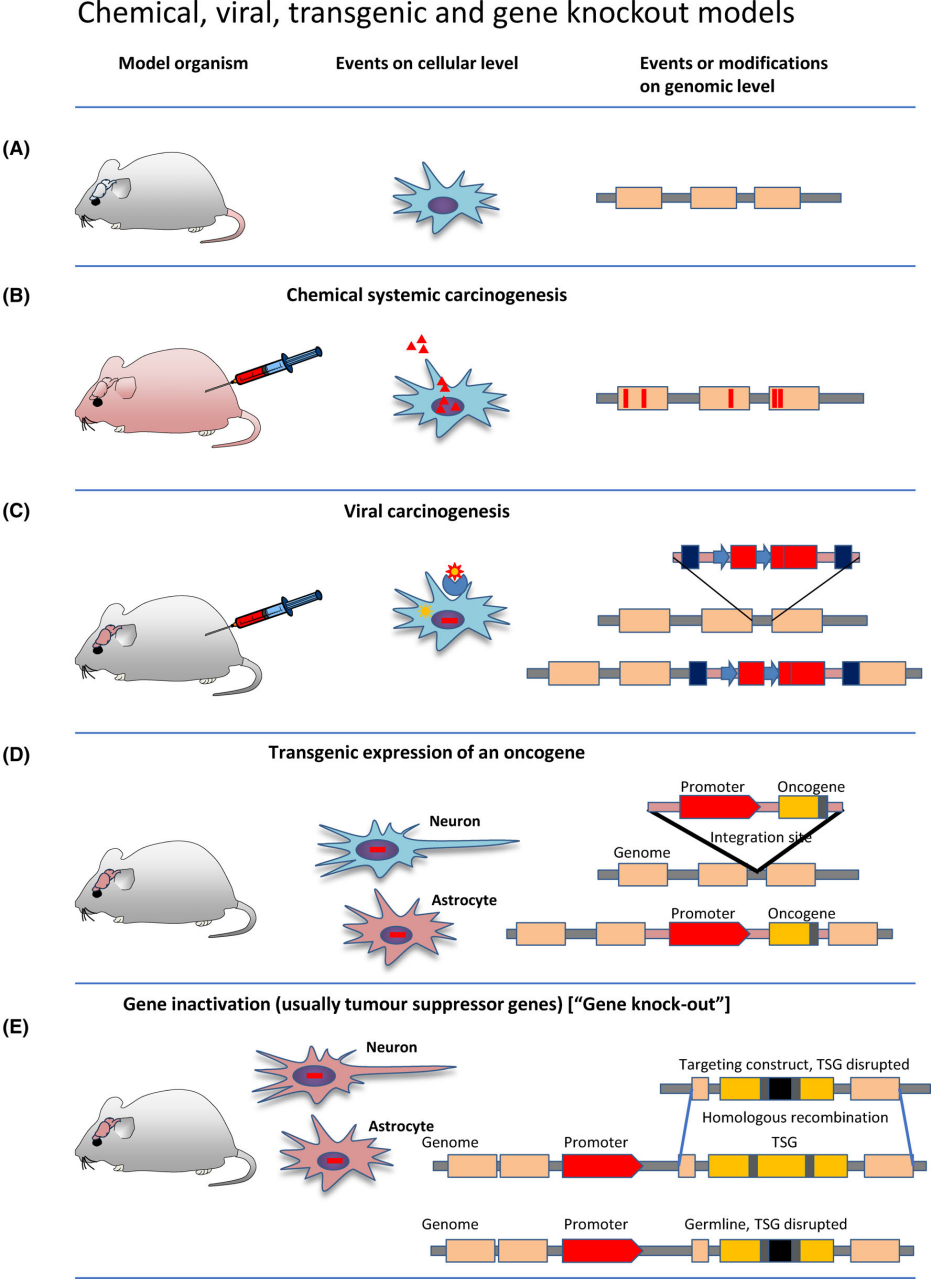
Brain tumour models: chemical, viral and constitutive transgenic oncogenesis: The left column shows the experimental approach, the centre column the events on cellular level and the right column the events or modifications on genomic level. (A) Schematic view of the layout with model system, cellular events and genomic events. (B) Chemical systemic carcinogenesis. The organisms used for this experimental approach were mostly rats, and less commonly mice, and other rodents. Organotropic action depends on the combination of species, their genetic background and the carcinogen. On the cellular level, carcinogens are resolved by the cell and acts in the nucleus. On genomic level, the carcinogen generates point mutations. (C) Viral carcinogenesis. The model organisms were mostly hamsters, rats, mice, and other rodents. The organotropic action depends on the combination of species, their genetic background and the virus strain. On a cellular level, the virus docks onto cells using an existing receptor and replicates using the cell's machinery. On genomic level, retroviruses integrate into the genome and disrupt and potentially activate endogenous genes. In the case of RSV, the retrovirus expresses the oncogenic v‐src kinase. (D) Transgenic expression of an oncogene. This experimental approach was predominantly done in mice. The organotropic or cell specific action of the transgene depends on the promoter that drives the expression of the oncogenic protein. On a cellular level, whilst being integrated in all cells of the organism, the transgene is expressed only in cells in which the promoter is active. On genomic level, transgenes randomly integrate into the genome. The expression pattern can slightly vary depending on the integration site. (E) Gene knockout, using homologous recombination in embryonic stem cells. The gene inactivation affects all cells in the organism. It was a fundamental experimental approach to understand the function of many genes, however the targeted deletion of a majority of tumour suppressor genes, such as RB, PTEN, or APC led to embryonic lethality, and the system was very soon replaced by the refined tissue‐specific Cre‐lox system.

This review will highlight the key discoveries, following a historical timeline combined with thematically coherent modelling strategies. The corresponding illustrations provide vignettes of the methodologies and mechanisms (Fig. 1A). The limitations of the review are the omission of all xenografting approaches, as described elsewhere [1], and there will be only a limited discussion of the mechanistic aspects of the biology discovered with these models.

## 2 Induction of CNS tumours with systemic or local carcinogens

The spontaneous formation of CNS tumours in common laboratory animals (rodents) is practically non‐existent [2] and therefore, the first approaches to generate such tumours in the 1930s and 40s in mice were of immediate importance [3, 4]. The carcinogens such as dibenzanthracene and other polycyclic hydrocarbons were applied locally, i.e. small crystals or pellets were implanted directly into the brain [5]. Initially, experiments with guinea pigs, rabbits and rats were largely unsuccessful, but larger systematic studies in mice yielded intrinsic neoplasms with a latency of 10 months. The published histological figures best correspond histologically to oligodendroglioma, glioblastoma, or medulloblastoma, and some neoplasms that cannot be easily classified [6, 7, 8]. The efficacy of tumour induction was comparably high, reaching nearly 50% [7, 8]. The histological features of the tumours depended on the site of implantation. Placement of the carcinogen in the cerebellum resulted in medulloblastomas, into the ventricle wall caused ependymomas, and oligodendrogliomas arose from the white matter of the hemispheres. Tumours that involve the meninges were either similar to high‐grade meningiomas, or they were gliomas involving a meningeal reactions (similar to gliosarcoma). The frequency and speed of tumour induction resulted in a relatively high popularity of this method. For some reason, the generation of brain tumours appeared to be of particular interest.

In the subsequent decades (1950s and 1960s), further carcinogenic compounds were developed and were applied systemically (‘resorptive carcinogens’). The mechanism of action of these compounds was an initial metabolization in the liver [9] by hydroxylation and demethylation [10]. The carcinogenic action of these compounds is not restricted to the liver, but depending on the chemical structure, could include various organs such as oesophagus, kidney, bladder, lung, or nasal cavity. The most commonly used compounds were *N*‐nitroso chemicals, specifically were methylnitrosourea (MNU), dimethylnitrosourea (DMNU), trimethylnitrosourea (TMNU), and ethylnitrosourea (ENU) [11]. These experiments were predominantly performed in inbred rat strains. Tumours developing in the central nervous system included a spectrum of glial tumours with morphological resemblance to glioblastomas, oligodendroglioma, and astrocytoma [6, 12, 13] (Table 1, Fig. 1B). Review of these published studies indicates a much higher degree of nuclear and cellular pleomorphism than in the corresponding human tumour types. An intriguing feature was the development of frequent micro‐neoplasia, visible only upon microscopic examination [13], and these occurred together with microscopically visible tumours elsewhere in the CNS. These chemically induced tumours showed often a higher degree of cellular and nuclear pleomorphism than corresponding human tumours [12]. The observation of tumour development (with morphological features similar to oligodendroglioma) in the vicinity of the subventricular zone corresponds well with the contemporary hypothesis of the origin of many forms of gliomas from the neurogenic zone of the SVZ [12]. Early forms showed more typically monomorphic oligodendroglial phenotype whilst more advanced neoplasms had an it mixture of astrocytic cells [12]. The advantages of chemically induced carcinogenesis are the intact micro environment and blood–brain barrier, and the immune system remains intact. The disadvantage is the limited predictability and reproducibility of the formation of neoplasms. The limitations of cell lines derived from gliomas induced in rats by chemical carcinogenesis have been discussed in detail elsewhere [14].

**Table 1:** Overview of key model systems discussed in the article.

Genetic lesions	Technology	Latency	Tumour incidence	Tumour type (as described in the publication)	Time period	References
Point mutations, not characterised	Local application of carcinogen (polycyclic hydrocarbons)	300 days	50%	High‐grade intrinsic tumours (oligodendroglioma, glioblastoma, medulloblastoma, unclassified)	1930s–1960s	[3, 4, 5, 6, 7, 8]
Point mutations	Systemic, *N*‐nitroso chemicals, specifically were methylnitrosourea (MNU), dimethylnitrosourea (DMNU), trimethylnitrosourea (TMNU), and ethylnitrosourea (ENU)	200–400 days	30–50%	High‐grade intrinsic tumours (oligodendroglioma, glioblastoma, medulloblastoma, but also soft tissue tumours)	1950s–1960s	[6, 11, 12, 13]
Integration into genome	Viral infection (RSV, human adenovirus 12 (dogs, hamsters, rabbits))	Wide range (20–400 days)	80% (brain tumours)	Glioblastomas, astrocytoma	1960s–1970s	[17, 18, 19, 20, 21]
SV40 Large T; p53	Transgenic construct, global expression	150–300 days (SV40) 40–60 days (SV40; p53)	100%	Choroid plexus tumours	1980s	[29, 30, 31]
GFAP; SV40 large T	Transgenic construct; tissue‐specific promoter, viral oncogene	Not applicable	Low	Disseminated glioma precursors in CNS	1990s	[23]
MBP;c‐neu	Transgenic construct; tissue‐specific promoter, oncogene	Too small numbers	Not established	High‐grade gliomas, PNET (embryonal tumours)	1990s	[45]
GFAP; v‐src	Transgenic construct; tissue‐specific promoter, viral oncogene	4–20 weeks	50%	High‐grade astrocytomas	1990s	[50]
GFAP; v‐src; p53^−/+^ or Rb^−/+^	Transgenic construct; tissue‐specific promoter, viral oncogene; tumour suppressor gene	Not provided	Not provided	High‐grade astrocytoma/glioblastomas	1990s	[52]
Ptc^−/+^	Tumour suppressor gene haploinsufficiency	25 weeks	20%	Medulloblastoma	1990s	[71]
GFAP‐TVA; Nestin‐TVA INK4a^−/−^	RCAS – tv‐a; tumour suppressor haplo‐insufficiency	3–8 weeks	30–50%	High‐grade gliomas/glioblastoma	1990s	[77, 80]
GFAP‐TVA, Nestin‐TVA; p16Ink4a^−/−^, p19Arf^−/−^	RCAS – tv‐a; tumour suppressor haplo‐insufficiency	6–10 weeks	50–80%	High‐grade glioma/lox	2000s	[87]
Nestin‐TVA; Shh, MYC	RCAS – tv‐a; oncogene	4–8 weeks	50–80%	Medulloblastomas	2000s	[94]
Nestin‐TVA; Ras, Akt	RCAS – tv‐a; inducible oncogene	4–8 weeks	50%	High‐grade glioma/glioblastoma	2010s	[98]
Nestin‐TVA; BRAF kinase^mut^	RCAS – tv‐a; inducible oncogene	Not provided	90%	Pilocytic astrocytoma	2010s	[99]
Nestin‐TVA; Akt, kras; (Ink4a/Arf^lox/lox^)	RCAS – tv‐a; Cre‐lox system	9 weeks	20–30%	Glioblastoma	2010s	[84]
Nestin‐TVA; R26‐LSL Cas9; PDGFB; sgRNA CDKN2A, sgRNA p53, sgRNA PTEN	RCAS – tv‐a; Cre‐lox system, CRISPR Cas9 system	4–8 weeks	50–100%	Glioblastoma	2010s	[109]
Nestin‐TVA; P53^−/−^; Ink4a/Arf^−/−^	RCAS – tv‐a; Cre‐lox system, CRISPR Cas9 system	5–8 weeks	90–100%	Astrocytoma, IDH mutant	2010s	[112]
Nestin‐TVA; IDH1R132H; ATRX^lox/lox^; PTEN^lox/loc^	RCAS – tv‐a; Cre‐lox system	6–12 weeks	90%	Astrocytoma, IDH mutant	2010s	[113]
GFAP‐Cre; Rb lox, p53 lox	Cre‐lox	12–15 weeks	100%	Medulloblastoma	2000s	[127]
GFAP‐Cre; Pten lox, p53 lox	Cre‐lox	25–50 weeks	100%	Glioblastoma	2000	[133, 209]
GFAcre; Nf1 flox	Cre‐lox	30+ weeks	20%	Glial proliferations	2000s	[134]
Nestin‐cre‐ERT2; Nf1 flox;p53flox; Pten flox/+	Inducible Cre‐Lox	20–60 weeks, depending on induction age	100%	Glioblastoma	2000s	[139]
Atoh1‐Cre; Blbp‐Cre Ctnnb flox(ex3); Tp53^flx/flx^; Lig4^flx/flx^ Xrcc2^flx/flx^ Ptch1^+/−^; Ink4c^−/−^	Cre‐lox	10–70 depending on genotype	20–100%	Medulloblastoma	2000s	[152, 153]
Nestin‐creERT2 or NG2‐creERTM; Nf1flox;p53flox;Pten flox	Inducible Cre‐Lox	40–60 weeks	100%	Glioblastoma	2010s	[137]
IDH1 R132H, p53 R270H, LSL‐Cas9‐GFP; sgATRX	Lentiviral Cre‐Lox CRISPR Cas	40–70 weeks	20–80%	Astrocytoma, IDH mutant	2020s	[198]
β‐Actin‐Cre; Nestin‐Cre Ptch1, Trp53, Pten; R26‐lsl‐SB11, Math1‐SB11; T2/Onc, T2/Onc2, T2/Onc3	Sleeping beauty	50–100 weeks	50–100% depending on genotype	Medulloblastoma	2010	[192, 210]
Ptch^+/−^, SB100/SB68, T2Onc	Sleeping beauty	25–40 weeks	90–100%	Medulloblastoma	2010s	[189]
Trp53R172H; Nes‐cre; T2/Onc2; SBase	Sleeping beauty	16–50 weeks	50–100%	Medulloblastoma	2010s	[188]
Tp53mut/SB11/T2Onc	Sleeping beauty	16–50 weeks	30–100% depending on genotype	Medulloblastoma	2010s	[186]
PDGF, PTEN, RB, p53, IDH R132H	Lentiviral gene transfer, cre‐mediated recombination, floxed tumour suppressor genes	4–10 weeks	80–100%	High‐grade gliomas/glioblastoma	2010s	[158, 159, 160, 161, 162]
PTEN, RB, p53	Adenoviral cre delivery, floxed tumour suppressor genes	40–80 weeks	10–20%	High‐grade gliomas/glioblastoma	2000s	[140]

## 3 Induction of CNS tumours by viral oncogenesis

The oncogenic potential of human adenovirus 12 was discovered in the 1960s [15] but only extrinsic tumours were generated, with high efficiency [15, 16]. Instead, a variety of strains of the Rous sarcoma virus (RSV) was generated and found to be more effective in inducing CNS tumours in dogs and to a lesser extent in rodents including mice. RSV has taken up the src gene, the first retroviral oncogene to be discovered and incorporated it into its genome, conferring the ability to stimulate and controlled mitotic activity of host cells. Intracerebral inoculation into hamsters [17], dogs [18], and rabbits [19] resulted in gliomas and choroid plexus papillomas. Injection of human adenovirus 12 into the brain of newborn hamsters or rats resulted in supratentorial embryonal tumours with formation of rosettes (at the time described as medulloepithelioma) [20]. The intracerebral injection of Rous sarcoma virus into young adult mice generated tumours described as ‘glioblastoma, astrocytoma and their variants’ [21]. Intracerebral inoculation of simian virus 40 (SV40) into newborn hamsters generated choroid plexus carcinomas (reviewed in [22]) (Table 1, Fig. 1C). The induction of choroid plexus tumours by SV40 resulted in a series of subsequent experiments expressing the SV40 large T antigen as transgene [23, 24, 25] (see below), and its detection in human ependymomas and choroid plexus papillomas [25, 26, 27] triggered a long‐standing debate of the possible viral origin of these tumours in humans, at the time thought to be proven by a detection of viral genomes in such tumours, but this association could not be subsequently verified [28].

## 4 Induction of CNS tumours in genetically modified mice

A new era of experimental brain tumour research started with the availability of transgenic technology, i.e. introduction of a gene of interest driven by a system or tissue‐specific promoter into the germline of a mouse, via injection into fertilised eggs [29, 30]. The first transgenic model of a brain tumour was engineered by expressing the SV40 early region, encoding the large and small T antigens, under the control of a metallothionein promoter/enhancer [31] (Table 1, Fig. 1D). Injections of two modifications of this construct into fertilised eggs resulted in the formation of malignant choroid plexus tumours, best characterised as choroid plexus carcinoma. Previous attempts by injecting SV40 DNA into the blastocoel cavity of mouse embryos did not result in the formation of tumours, possibly due to an incomplete mosaicism of the animals generated [32]. The formation of choroid plexus tumours in the transgenic animals raised an important biological question, as to why the SV40 T antigen was active in choroid plexus epithelial cells, but not in other progenitor or mature cells in the CNS. Of the 95 pups born, 25 were transgene carrier, developing brain tumours, but also liver, kidney and muscle tumours. Within the CNS, all animals had choroid plexus tumours only, making it unlikely that the integration site is responsible for the observed tropism of the oncogenic activity, but suggests that the choroid plexus (progenitors) are the most permissive cell type for T antigen activation. The success and the high efficacy of the tumour induction using the SV40 large T antigen, rendered this system as a tool with high utility to study carcinogenesis including tumours of the CNS. The SV40 large T antigen binds to, and suppresses the protein product of the tumour suppressor genes *Rb* [33, 34] and *p53* [35], and other members of the pocket protein family, such as *p107* and *p130* [34, 36, 37]. Subsequently, the role of the tumour suppressor p53 was further dissected by generating SV40 variants with ablated *p53* suppression, in the context of genetically modified *p53* heterozygous or *p53* null mice [38, 39].

The consecutive refinement of transgenic technology, in conjunction with a rapid progress in identifying oncogenes, resulted in a much higher flexibility of combination of promoters and oncogenes [40, 41, 42]. Instead of using viral promoters with poorly defined tissue specificity [43, 44], the following transgenic mouse models expressed potent oncogenes under the control of a cell‐ or tissue‐specific promoter to direct oncogenesis into cells that were at the time thought to be the origin of brain tumours. The *Gfap* promoter was used to induce astrocytoma (Fig. 1D), and myelin basic protein as a promoter with the aim to generate oligodendrogliomas. For example, the c‐neu oncogene (tyrosine kinase), expressed under the control of a myelin basic protein promoter, directed the oncogene expression to mature oligodendrocytes [45]. With hindsight this was however not an ideal cell type, as oligodendrogliomas are more likely to originate from NG2 populations of stem and progenitor cells. Indeed, *Mbp‐c neu* transgenic mice developed primitive neuroectodermal tumours, with Gfap and neurofilament expressing, highly pleomorphic cells, which had no resemblance to oligodendrogliomas [45]. Likewise, transgenic mice expressing the SV40 large T antigen under the control of the human (*GFAP*) or murine *Gfap* promoter exhibited a proliferation of cells in the periventricular subependymal zone, leading to early lethality. The neoplastic cell populations had a primitive, uniform morphology, and infiltrated adjacent developing CNS tissue with formation of secondary structures around blood vessels and neurons [23]. Passaging such tumour cells as allograft into immunocompromised mature mice (nude mice) retained this growth pattern. The localisation of micro‐neoplasms and fully developed primary tumours in the subventricular zone, and in other areas of neurogenesis (such as the hippocampus) [23] can be explained by discoveries that were only emerging at that time: Not only mature astrocytes (the intended target of the transgene expression), but also neural stem and progenitor cells express GFAP [46, 47], thus explaining the neoplastic transformation within cell populations of the germinal matrix in newborns.

A further modification of the concept of combining a tissue‐specific promoter with an oncogene to generate CNS tumours was the expression of the viral oncogene *v‐src* under the control of the *Gfap* promoter. Src (pp60c‐src) is an intracellular tyrosine kinase expressed ubiquitously in mammalian cells, with highest levels encountered in brain and platelets [48]. *v‐src*, the transforming gene of the Rous Sarcoma Virus (RSV) encodes pp60v‐src which lacks the carboxy‐terminal region comprising Tyr‐527 (which switches off the kinase activity), resulting pp60v‐src to be constitutively active [49]. 20% of *Gfap; v‐src* mice developed multifocal astrocytomas at 4 weeks of age [50]. Similar to *Gfap‐SV40* transgenic mice, the oncogenic signal is targeted to cells expressing GFAP, which includes mature astrocytes [50]. The additional deletion of the tumour suppressor gene *p53* however did not accelerate tumour development or increase incidence [51]. This model did recapitulate typical features of high‐grade gliomas, including the development of microvascular proliferations [52], but it does not formally address the biological question of the cell of origin of intrinsic brain tumours. Firstly, the *GFAP* promoter is not only expressed in astrocytes (which was the prevailing hypothesis at the time [53]) but is also transiently expressed in progenitor cells throughout the CNS [54]. Therefore, models using such constructs could not pinpoint the cell of origin. In this model discussed here, it is therefore possible that the *Gfap‐v‐src* expression may have triggered neoplastic transformation of stem or progenitor cells. This approach, even though creating high‐grade gliomas with high efficacy and good morphological resemblance to high‐grade gliomas, was soon replaced by more refined models [55], using advanced technologies such as the conditional gene inactivation using the Cre‐lox system [56], adapted to mouse transgenesis in the mid‐1990s [57].

## 5 CNS tumours in haploinsufficient mice

The discovery of the involvement of tumour suppressor genes such as for example *RB* [58], *p53* [59, 60, 61], *PTEN* [62, 63, 64] in glial neoplasms including glioblastomas, or genes involved in the sonic hedgehog signalling (*SHH*, *PTCH*, *GLI*, *SUFU* [65, 66]) or the *wnt* signalling pathway (*beta‐catenin*, [67], *APC* [68]) predominantly in medulloblastomas but also other CNS neoplasms, alongside with the discovery and characterisation of some of these genes in Drosophila prompted the generation of deletion mutants in mice, before the *Cre‐lox* technology was either established or more widely adopted. The majority of mice with homozygous deletion of such tumour suppressor genes showed severe phenotypes, often with embryonic lethality, whilst the heterozygous mutants develop neoplasms including in the CNS, for example pituitary tumours in *Rb‐*heterozygous mice [69, 70], medulloblastomas in *Ptch* [71] or *Sufu* [72] heterozygous mice. Instead, no CNS tumours were found in heterozygous *p53* mutants (soft tissue sarcomas and haematological neoplasms) [73], *Apc* (colorectal tumours, [74, 75]), or *Pten* (Lymphomas, dysplastic intestinal polyps, endometrial complex atypical hyperplasia, prostatic intraepithelial neoplasia, and thyroid neoplasms [76]).

## 6 CNS tumours generated by combining virus receptor expression and engineered viruses expressing activated oncogenes: The RCAS – tv‐a model

### 6.1 History of the model system

In the late 1990s, the laboratory of Harold Varmus generated a novel system that enables flexible delivery of oncogenes via retroviral vectors into transgenic mice expressing receptors for these viruses under a cell‐specific promoter [77]. At the same time, the characterisation of genetic alterations in human gliomas had made significant progress: it was established that glioblastomas were characterised by different combinations of mutations of the tumour suppressors *PTEN* [62], *p53* [61], *RB* [58], deletions in the *INK4a* locus [78], thus being unable to make the two *INK4a–ARF* gene products, *p16INK4a* and *p19ARF*, which arrest the cell, or the amplification of *CDK4* [78] or epidermal growth factor receptor (*EGFR*) [79]. This knowledge provided a strong, evidence‐based rationale for the generation of model systems, based on the combination of a transgenic mouse, which expresses the virus receptor TVA (the receptor for avian leucosis virus‐subgroup A, ALV‐A), under the control of a promoter directing expression to designated cell types of the CNS, such as astrocytes (GFAP, i.e the *GFAP* promoter driving expression of the quail *tv‐a* cDNA, in short *Gtv‐a*) or in neural stem cells (nestin, in short *Ntv‐a*). The virus component was then genetically engineered to express genes of interest, such as *FGF*, *EGFR*, *CDK4*, and including reporter genes such as alkaline phosphatase enabling histological identification [77, 80].

Using this system, a proof‐of‐principle experiment, performed with *in vitro* engineered cells, transplanted into a recipient mouse brain confirmed that *ex‐vivo* cultured mouse astrocytes expressing GFAP‐tv‐a can be infected with ALV expressing basic fibroblast growth factor [80]. These cells grow *in vitro*, proliferated and migrated and also formed small groups of transformed astrocytes, but failed to form tumours [80]. Instead, a more aggressive phenotype was observed in cultures expressing CDK4 in astrocytes: in this setting, transformed astrocytes grew rapidly and became immortalised, similar to astrocytes with a deletion of the INK4A locus [81].

### 6.2 The RCAS‐TVA model and oncogene expression

Following this proof of principle *in vitro*, the system was then applied *in vivo*. Transgenic mice expressing the *tv‐a* receptor (henceforth TVA) under control of the GFAP (*GTVA*) or the Nestin promoter (*NTVA*) were inoculated with a virus constructed to express a constitutively active, mutant form of human *EGFR* with deletions of intra‐ and extracellular sequences (termed *RCAS* (Replication‐Competent Avian Sarcoma‐Leukosis Virus Long Terminal Repeat with a Splice Acceptor)‐EGFR* by the authors), or *RCAS‐Cdk4*. *GTVA* and *NTVA* mice, transduced with EGFR* did not develop tumours, only further crossing into an *Ink4a* heterozygous or *Ink4a* null background increased the tumour incidence to nearly 50%. Further transduction with *Cdk4* and *bFGF* expressing viruses increased the incidence to 10% in the *Ink4a* wild‐type background. Instead, ablation of *p53* had no significant effect [82]. The efficacy of gliomagenesis was higher in NTVA mice than in GTVA mice, presumably due to a higher susceptibility and/or higher abundance of stem/progenitor cells expressing nestin than the populations expressing GFAP either during their stem cell differentiation status or the reduced susceptibility of terminally differentiated astrocytes.

Subsequently, the discovery of further pathways involved in gliomagenesis, such as the *ras* and *PTEN/Akt* pathways, this system showed its versatility, in that the same transgenic *GTVA* and *NTVA* mice were used as host, whilst the virus was modified, to express the G12D variant form of *k‐ras* (*RCAS‐kras*), or a constitutively active variant of Akt (Akt‐Myr ∆11–60 [83]). The expression of *kras* or *akt* alone did not generate tumours, whilst their combination elicited high‐grade glioma in 25% of *NTVA*, but not in *GTVA* mice [84]. This again underpinned the higher susceptibility of mice expressing the virus receptor in a nestin‐expressing population (Ntv‐a) than in GFAP‐expressing cells (*GTVA*). A further development and refinement of the system was the crossing Gtv‐a or Nestin‐Tva (Ntv‐a) mice into either a *p16Ink4a* ^−/−^ [85] or the *p19Arf* ^−/−^ [86] background. Transduction of Nestin‐expressing cells was generally more effective than targeting GFAP‐expressing cells, and the combination of *Ras* and *Akt* was more effective than expression of *Akt* or *Ras* alone. The highest rate of glioblastomas (83%) was seen in *GTVA* mice transduced with *Akt* and *k‐ras*, in an *Arf* ^−/−^ background [87].

In contrast to the MAP kinase‐induced tumours with variable, sometimes distinctive glial differentiation, tumours induced by PDGF show an oligodendroglial phenotype [88]. One of these models combined the *NTVA* and *Ink4a/ARF* mouse lines with RCAS viral vectors encoding PDGFB [89] and IGFBP2 [90]. Although IGFBP2 by itself does not result in cancer development, only one additional oncogenic event, such as K‐Ras or PDGFB induces gliomas. Another model generating oligodendroglioma‐like tumours used the 20,30 cyclic nucleotide 30‐phosphodiesterase (CNP) promoter (directing expression to myelinating oligodendrocyte progenitor cells (OPC), as well as Schwann cells), to express the viral receptor in transgenic mice. The expression of PDGFB in OPCs induced gliomas within distinct oligodendroglial (i.e. clear cell) appearance with an incidence of approximately 30% [91].

Medulloblastomas frequently have either an activation of the *wnt* signalling pathway (wnt‐type of medulloblastoma) or a dysregulation of the sonic hedgehog (*SHH*) pathway. Here, the TVA model was used to express NTVA in a heterozygous background of *Ptc* (+/−), which are prone to develop medulloblastomas. These mice were inoculated with the RCAS virus expressing‐*SHH*, *MYC*, or both. The tumour induction rate was between 20% and 40%, and there was no significant effect of the *Ptc* allele loss, and there was no significant effect of the MYC expression [92]. A follow‐up study utilised IGF2 signalling and Akt (Akt‐Myr‐D11‐60) activation, creating medulloblastoma with approximately 50% incidence when combining RCAS *Shh* and *IGF2* or *Shh* and *IGF2*, in NTVA mice in a *Ptc* wild‐type background [93]. A similar study elicited medulloblastomas in NTVA mice inoculated with RCAS *Shh*, *Gli*, *IGF2*, *N‐myc*, or *N‐myc* T50A. In this model, virus‐producing cells were injected in the cerebellum of newborn mice, to enhance and extend virus production. Only RCAS‐*Shh* or RCAS‐*Shh* + *MYC* or RCAS‐*Shh* + *MYC T50A* virus elicited medulloblastomas, whilst all other combinations resulted in no tumourigenesis [94]. Subsequent medulloblastoma models further explored other pathways which had been identified in human medulloblastoma, such as BCL2 (combining *Bcl2* and *Shh* expression in the background of NTVA mice, resulting in nearly 80% tumour induction rate [95]), and also a high frequency (80%) of medulloblastoma formation in mice after postnatal expression of hepatocyte growth factor, the ligand of the *met* receptor in cooperation with Shh [96].

### 6.3 The RCAS – tv‐a model and inducible oncogene expression

In a further modification of this successful, versatile model, Holmen and Williams [97] injected Ntv‐a mice with ras, Akt, and tet on/off vectors. The tet on/off vector allows for a Doxycycline‐inducible activation of the transgene, i.e. expression of Ras and Akt. Without Doxycyclin administration, mice do not develop any tumours, but once the tet system is activated by injection of doxycycline for 4–6 weeks, Ras and Akt gene expression is activated, and in the context of this model results in formation of glioblastoma with an incidence of nearly 50%. More recently, the same group used the same system to express Activated Mek (MAPK/extracellular signal‐regulated kinase (ERK) kinase (MEK), a RAF effector), to induce tumours *in vivo* in the context of activated Akt or INK4a/Arf loss, showing that indeed activated MEK cooperates with Ink4a/Arf loss or Akt activation to induce gliomas *in vivo* [98]. Likewise, activated (V600E mutant) BRAF again in the context of INK4a loss, result in a high frequency of malignant gliomas, but also in poorly differentiated intrinsic tumours [98]. In human brain tumours, BRAF V600E mutations are seen in 5% of posterior fossa pilocytic astrocytomas, approximately one third of gangliogliomas and nearly 70% of pleomorphic xanthoastrocytomas, and a small proportion of glioblastomas. In refinement to study the role of other BRAF alterations, Ntv‐a mice were inoculated with viral construct expressing for variance of a BRAF construct, the full‐length gene (BRAF WT FL), the full‐length gene carrying the V600E mutation (BRAF VE FL), the WT kinase domain spanning exons 9–18 of WT BRAF (BRAF WT kin), and the V600E mutated BRAF kinase domain (BRAF VE kin). When injected into the posterior fossa, only the BRAF VE kin elicited tumours with a strong resemblance to pilocytic astrocytomas [99].

### 6.4 The RCAS – tv‐a model combined with Cre‐lox technology

**Fig. 2:**
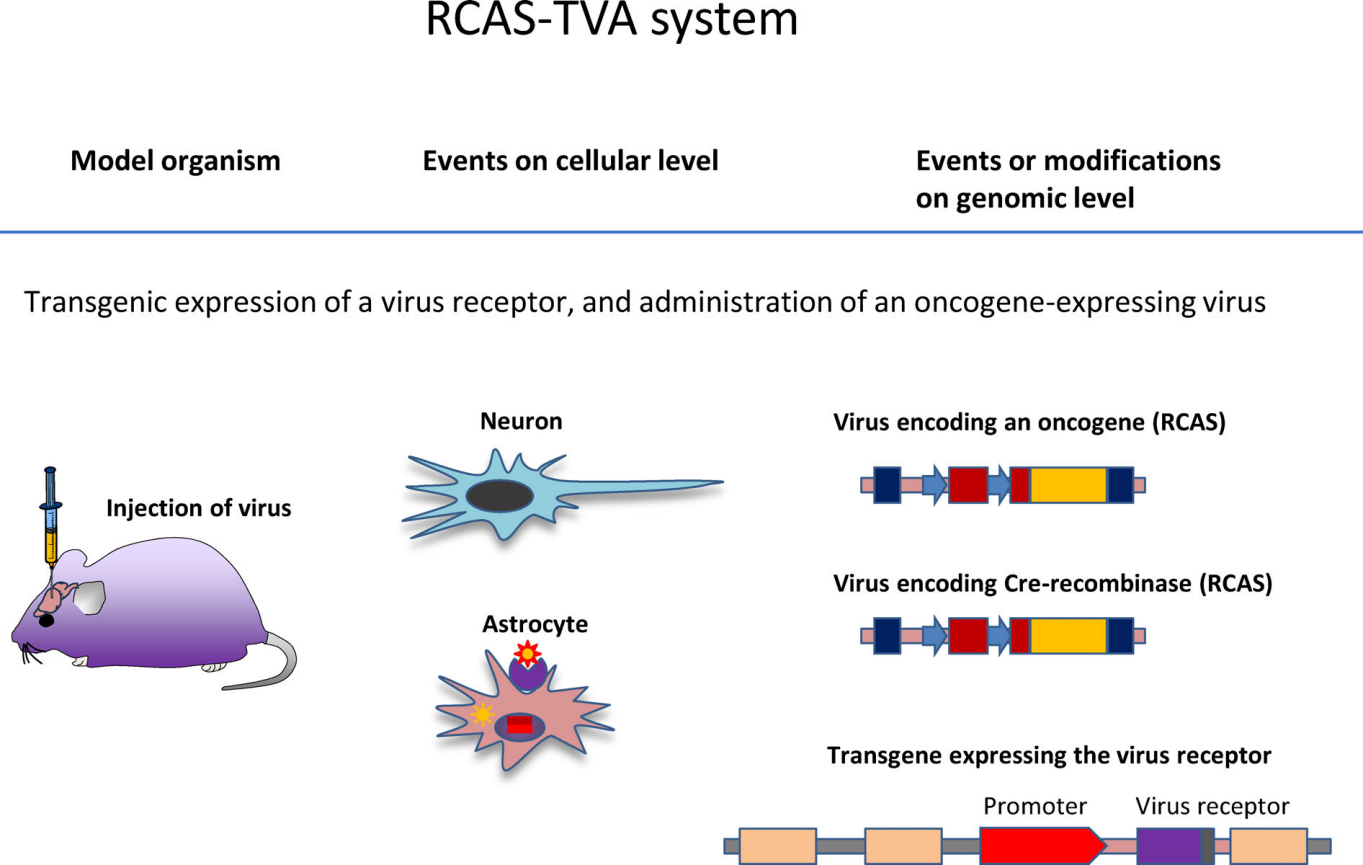
Brain tumour models, the RCAS‐TVA system: the only organism used in this setup were mice. The organotropic or cell specific action depends on the promoter that drives the expression of the receptor. The oncogenic action depends on the engineering of the virus. On a cellular level, the mechanism of action is a transgenic expression of a virus receptor on a desired cell type, in most brain tumour models this were *GFAP* or *Nestin* promoters. The virus is genetically engineered to match the receptor. On genomic level, the transgene expression the virus receptor is integrated in all cells of the genome, but only cells in which the promoter is active (for example astrocytes for the GFAP‐TVA transgene), express the receptor. The virus is injected into the transgenic mouse, and docks onto the receptor, determining the delivery of oncogenes or, in modified experimental setup, CRISPR Cas9 constructs or Cre‐recombinase.

**Fig. 3:**
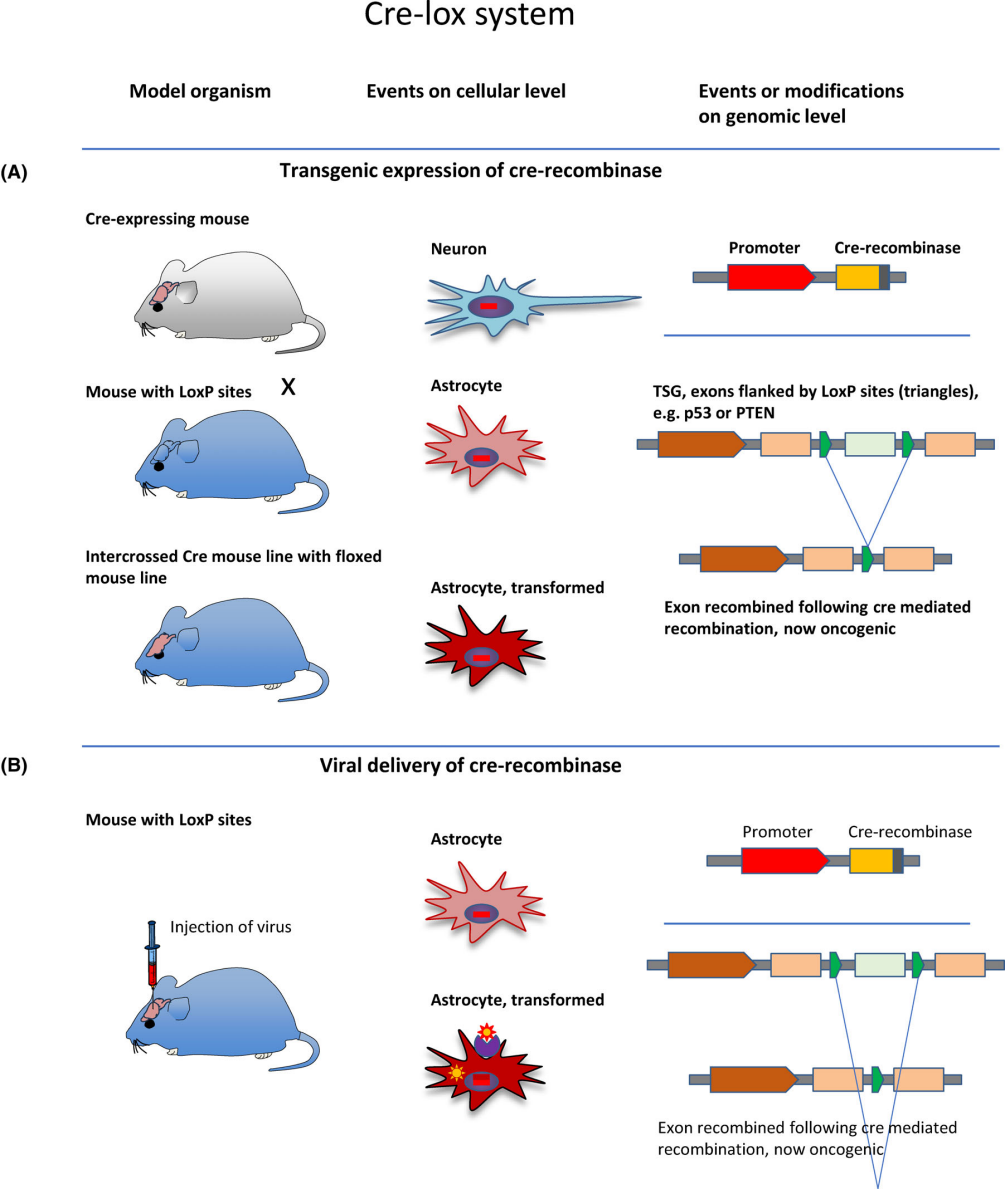
Brain tumour models, the Cre‐lox system: (A) One mouse line is generated to express a Cre‐recombinase. The organotropic or cell specific action depends on the promoter that drives Cre expression. A second mouse line is generated with *loxP* recognition sites flanking a gene of interest (‘floxed’), and in the context of brain tumour modelling, these were typically tumour suppressor genes such as *p53*, *Rb*, *Pten*. On cellular level, the *Cre* transgene is integrated in all cells of the organism, but are expressed only in cells (transiently or permanently) in which the corresponding promoter is active. In this example, only GFAP‐expressing cells are targeted, i.e. the tumour suppressor gene is recombined only in these cells, notably this can occur also transiently during development. On genomic level, the transgenically expressed Cre‐recombinase recognises pairs of *loxP* recognition sequences, and through the formation of a loop, the DNA stretch between two *loxP* sites is removed. One *loxP* site remains in the genome. (B) A modified approach (used to target specific regions of cell types that cannot be easily targeted by transgene expression) uses a conditional knockout mouse (‘floxed mouse’), into which a virus expressing Cre‐recombinase is injected in the targeted fashion.

The availability of the Cre‐lox technology was also harnessed for the TVA system (Fig. 2), with a model expressing activated forms of BRAF (V600E), K‐ras (G12D), as well as *Akt*, and Cre‐recombinase (Fig. 3). The model also carried conditional *Ink4a/Arf* alleles (*Ink4a/Arf* ^*lox/lox*^), which were then recombined by the activity of Cre‐recombinase. Interestingly, neither did the expression of BRAF V600E alone in the targeted stem/progenitor subpopulation result in the formation of intrinsic neoplasms, nor did the isolated loss of *Ink4a/Arf*; instead, the combined expression of BRAF V600E and loss of *Ink4a/Arf*, led to the development of poorly differentiated intrinsic neoplasms (incidence approximately 40%), with no glial marker expression. In contrast, the combined expression of BRAF V600E and Akt (in an *Ink4a/Arf* wild‐type context) resulted in formation of pleomorphic glial tumours at a slightly higher frequency (nearly 50%). The expression of K‐ras (G12D) alone also did not result in formation of intrinsic neoplasms, as previously shown [84], but required the additional expression of activated AKT or the loss of *INK4a/Arf*, resulting in approximately 50% incidence of glial tumours. A further model combined the Cre‐lox technology with *tet* inducible technology. As before, NTVA transgenic mice were used to direct RCAS expression to nestin‐expressing progenitors. This study examined the dependence of gliomas on continuous KRas signalling in the context of *Ink4a/Arf* deficiency. Doxycycline was administered to suppress KRas expression, with survival for doxycycline‐treated mice increasing than that for untreated mice [100].

### 6.5 The RCAS – tv‐a model combined with Cre‐lox and CRISPR Cas9 technologies

**Fig. 4:**
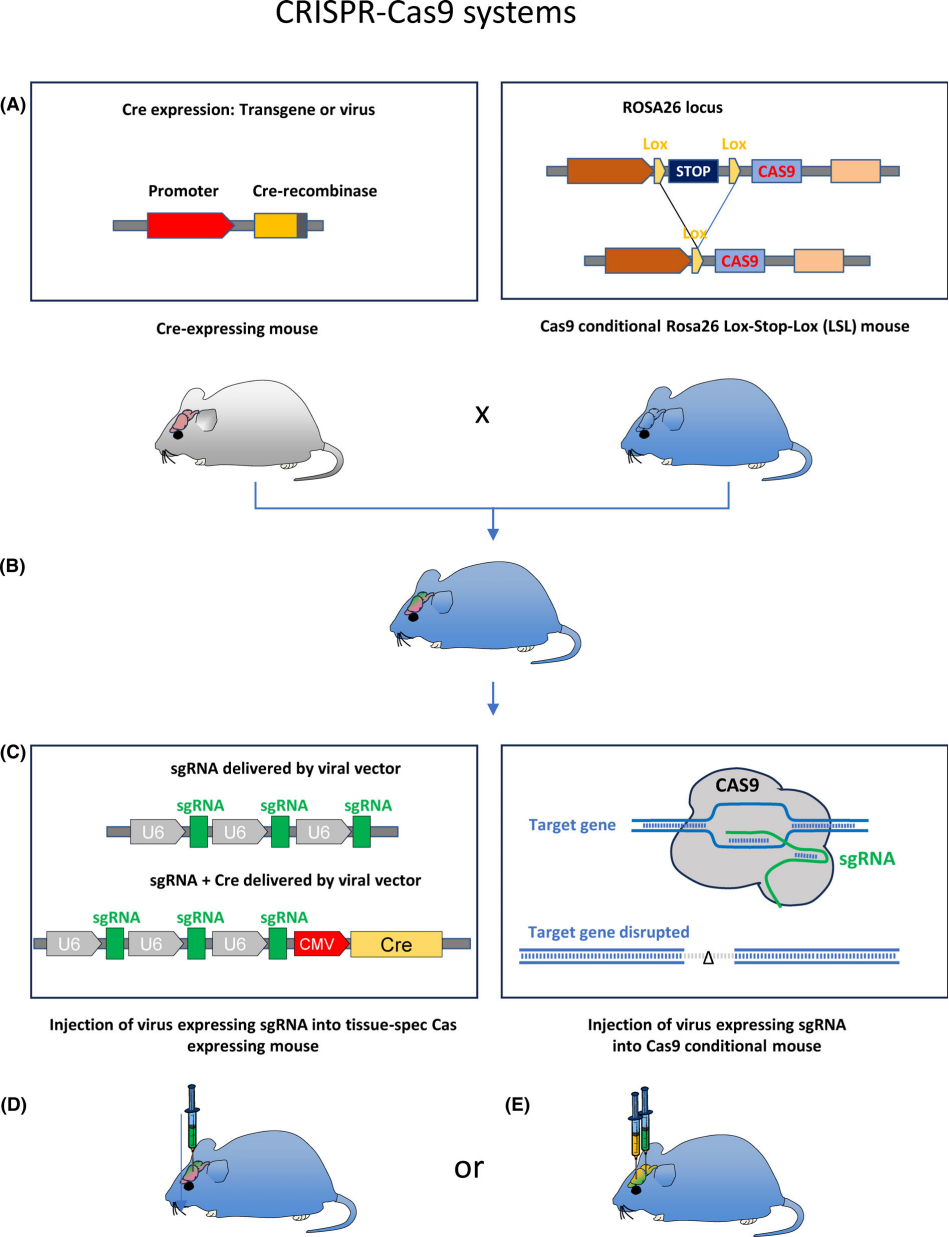
Brain tumour models, CRISPR Cas9 system: somatic mutagenesis using the CRISPR Cas9 technology can be used in different methodological approaches, involving two (or more) components. One component comprises the delivery of a single guide RNA (sgRNA), leading to the introduction of a deletion in the desired target genes. The sgRNA directs the Cas9 nuclease to a complementary sequence in the genome where Cas9 induces a double‐strand break. Cas9 can either be expressed via delivery by a viral vector, or expressed by the host, either constitutively, or conditionally by Cre‐mediated expression in the *Rosa26 lox‐stop‐lox* model. The Cre‐expressing mouse is intercrossed with the *Cas9 Rosa26 LSL* mouse, resulting in the expression of Cas9 in the desired cell population of the CNS. This mouse is then injected with a virus expressing sgRNA, resulting in a tissue‐specific inactivation of the desired target genes. (A) Intercrossing the mouse line expressing Cre‐recombinase under a control of a cell‐ or tissue‐specific promoter with the *Cas9 Rosa26 LSL* mouse results in the Cas9 expression in the desired target cell population (B). Subsequently, a viral construct expressing guide RNA (C) is delivered to the brain (D), where it disrupts the gene of interest in Cas9 expressing cells. Alternatively, the sgRNA construct can be engineered to also express Cre‐recombinase (D) and delivered directly into a Cas9 Rosa 26 LSL mouse (E). The CRISPR Cas9 system can also be combined with the RCAS‐TVA or the sleeping beauty/PiggyBAC system.

The availability of high‐throughput technologies to interrogate the genomic landscape of cancer led to the discovery of not only common driver mutations, but increasingly rare mutations including fusion events. This demands an adaptation of mouse models, to respond to the increasing need of a rapid and flexible gene editing to establish the role of such mutations, and importantly, to generate realistic preclinical models, for example for a drug testing. The CRISPR (clustered regularly interspaced short palindromic repeats) Cas9 system, a gene editing technology, has made it possible to manipulate nearly any gene candidate *in vivo* accurately and in a highly targeted fashion (Fig. 4). The system offers sequence‐specific direct editing of DNA and therefore, in contrast to RNA‐interference‐based approaches, this method can achieve complete loss of function of the encoded protein. In the context of experimental models of cancer, it allows inactivation of tumour suppressor genes [101, 102, 103], generation of somatic variants [101, 102, 104], and even more complex genomic rearrangements including fusion events [105, 106]. To respond to these demands for a flexible preclinical model, a sophisticated integration of the RCAS‐TVA (Fig. 2), Cre‐lox (Fig. 3) and CRISPR Cas9 (Fig. 4) technologies has been developed. This effectively creates a mouse model combining the gene editing capability of the CRISPR Cas9 system with the speed and simplicity of somatic gene delivery of the RCAS‐TVA approach. The *Rosa26‐LSL Cas9* (LSL‐Cas9) knock‐in mouse strain [101] was intercrossed with the transgenic *NTVA* or *GTVA* mouse lines [81]. Whilst previously RCAS‐Cre‐expressing plasmids were used in combination with TVA‐expressing mice to achieve tissue‐specific deletion of a variety of floxed alleles [107, 108], this model [109] achieved the Cre delivery instead by intercrossing of *Nestin‐Cre* [110] or *GFAP‐Cre* [111] mice.

The first model of an IDH‐mutant astrocytic tumour using the TVA system combines the NTVA model to deliver mutant IDH1 (R132H) into nestin‐expressing progenitor cells (i.e. a surplus to the existing alleles instead of a replacement). Both glioma types were driven by PDGF in combination with p53 loss, in keeping with the p53 mutation in IDH‐mutant astrocytomas. However, unlike in human astrocytomas, this mouse model was not engineered to have loss of ATRX expression. Also, these mice had additional deletions of *Ink4a/Arf* (i.e., *NTVA; Ink4a/Arf* ^−/−^; ^+/−^ or ^+/+^), and unsurprisingly, *Ink4a/Arf* deficiency correlates with formation of higher grade gliomas both, in humans and mice [112]. A subsequent model using the same technology was more realistic, in that it recapitulated the ATRX loss of function in IDH‐mutant astrocytomas, although this model does not specifically delete *p53* [113]. This model confirmed previous studies (using a different approach to express mutant IDH1 [114, 115]), in that the sole expression of mutant IDH1 in nestin‐expressing precursor cells does not lead to tumour formation in the lifetime of these mice (delivery of RCAS‐Cre and RCAS‐IDH1R132H into NTVA; *Cdkn2a* ^*lox/lox*^; *Atrx* ^*lox/lox*^; *Pten* ^*lox/lox*^ mice). Only the additional delivery of RCAS‐*PDGFA* led to a high incidence of 90% Astrocytoma development.

### 6.6 Conclusions and lessons learned from the TVA models

A detailed and comprehensive review of the utility of TVA models has highlighted the main advantages and limitations of this model system [116].

The principal advantages of this model system are:

- Multiple genetic alterations and their cooperation can be investigated in a single mouse line, and they can be combined with other model systems, such as the *Cre/loxP* system, knock‐in/knockout and transgenic mouse models.
- The gene transfer can be simultaneous or sequential and can be performed in a spatial–temporal manner.
- Similar to the availability of reporter mouse lines in the *Cre/loxP* setting, reporter genes can be introduced for lineage tracing and fate mapping. Genomic integration results in expression in all progenies.
- Low infection efficiency of some tissues (range < 1% to 20%), when the transgene is expressed only in a small number of cells within an organ.

The main limitations are:

- Cell division is essential for effective viral integration, i.e quiescent cells cannot be targeted.
- Relatively small insert size of the RCASBP(A) construct (2.8 kb).
- Low infection efficiency *in vivo* (< 1% to 20%). Low penetrance of the phenotype possible, which can be a disadvantage for tumour models.
- Possible immune reaction against the inserted gene product.
- Gene expression level is influenced by the integration site into the host genome, and a random integration into the host genome can influence expression of host genes.
- The RCASBP(A) virus can accumulate mutations/deletions after several rounds of virus replication in DF‐1 cells.
- Epigenetic silencing of the TVA transgene can occur in transgenic TVA lines generated by pronuclear injection, however this limitation applies to transgenic mouse lines generally.

Not all advantages or disadvantages are necessarily specific to this model but may represent a limitation also to other genetically modified mouse models, including knock‐in, knock‐out, and transgenic mouse lines.

## 7 The Cre‐lox System

### 7.1 Introduction and history of the Cre‐lox system

In the mid‐1990s, the technology of ‘conditional’ *in vivo* gene inactivation with the *Cre‐lox* system fundamentally changed the opportunities of disease modelling. Until then, inactivation of genes was mostly limited to the generation of a heterozygous or homozygous (constitutive) null‐mutant and could result in embryonic lethality. For example, embryonic lethality was observed in animals with constitutional ‘knockout’ for *RB* [70], *APC* [117, 118, 119], or *PTEN* [120, 121]. Although these models greatly contributed to the understanding of the function of the tumour suppressors, they did not allow the study of the role of these tumour suppressors in specific organ systems. The inducible gene targeting technology overcame this limitation. The gene of interest (usually one or several exons) is engineered to carry a 32 base pair *loxP* recognition sequence on either side (i.e., 5′ and 3′) of the region to be excised (simple schematic in Fig. 3). The excision of the *loxP* site is accomplished by the action of the enzyme Cre‐recombinase, which forms and excises a loop between the two LoxP sites. The result is the removal of the sequence flanked by the two sides and only a single *loxP* site remains in the genome [57, 122, 123].

The expression of Cre‐recombinase in the desired target cell population, tissue, organ can be achieved with several different methods: The most popular approach is the generation of a separate mouse line which expresses Cre‐recombinase under the control of a cell‐ or tissue‐specific promoter. This Cre‐expressing mouse is then crossed with the mouse carrying *loxP* sites. The result is a removal of the gene of interest (i.e. flanked by the loxP sites) in cells which express Cre‐recombinase transiently or permanently. The fact that even a transient expression of Cre‐recombinase can lead to the recombination of the target gene flanked by *loxP* sites (‘floxed’) is very important to understand and correctly interpret phenotypes in this model system. For example, in order to achieve a functional inactivation of the *PTEN* gene in cells expressing GFAP (i.e., astrocytes but also stem and progenitor cells) a *GFAP‐Cre*‐expressing mouse is crossed with a *Pten* floxed mouse, to obtain the *GFAP‐Cre; Pten* ^*loxP/loxP*^ genotype. This mouse was created to model *PTEN*‐deficient glial tumours, but instead a severe developmental phenotype with enlarged brains to a disturbance of the neural precursor migration, caused by *PTEN* loss in neural stem and progenitor cells [124, 125]. A more severe phenotype with embryonic lethality was observed in *Nestin‐Cre; Pten* ^*loxP/loxP*^ mice, due to a widespread deletion of *PTEN* in a wider range of neural progenitors [126].

Such early developmental expression of Cre‐recombinase was later circumvented with several methods: (a) restriction of the Cre expression, such as a region‐selective expression or expression under the control of promoters which are not developmentally expressed [127], (b) topical application or delivery of Cre‐recombinase, for example using an adenoviral vector [128, 129, 130], or (c) use of an inducible *Cre* transgene, for example in mice expressing the *Cre* transgene under the control of a cell‐specific promoter which has to be activated by the drug tamoxifen (Cre ER (T) system, [131, 132]).

### 7.2 Transgenic and inducible transgenic Cre expression: glioma models

Several studies show that the expression of Cre‐recombinase in astrocytes, neural progenitor cells and neural stem cells, in combination with conditional inactivation of *p53*, *Nf1*, *Pten*, *Rb* lead to the formation of high‐grade gliomas. Expression of Cre‐recombinase under the control of the human *GFAP* promoter in mice with combined recombination of *Pten* and *p53* (*Gfap‐Cre*, *PTEN* ^*lox/lox*^; *p53* ^*lox/lox*^) [133] causes the formation of diffuse high‐grade glioma, morphologically resembling human glioblastoma. In a similar approach, an *Nf1* mutation was activated during development in the neural stem/progenitor population by expression under the control of the human *GFAP* promoter, resulting in *GFAP‐Cre*, *Nf1* ^*lox/lox*^ mice. These mice developed a generalised gliosis but no obvious neoplastic lesions in the brain; however, there were features suggestive of optic pathway glioma in a proportion of these animals [134], consistent with the involvement of deregulated map kinase pathways in the pathogenesis of pilocytic astrocytomas [135]. A role of *NF1* was later also established for a subset of IDH‐wild‐type glioblastomas [136], confirming the relevance of the corresponding mouse model [137, 138]. The expression of the *GFAP‐Cre* transgene was refined in a study using the ER(T) inducible model system, by which the Cre‐recombinase is activated through a tamoxifen inducible system, i.e. an oestrogen receptor is transgenically expressed, and activates the downstream Cre‐recombinase upon systemic administration of tamoxifen. In this system, Cre‐recombinase is fused with a mutated oestrogen receptor (ER) domain. Normally (i.e. without tamoxifen), this ER domain remains inactive, due to Cre ER(T) complex sequestration, involving heat shock protein (HSP) 90 (a chaperone protein). Administration of tamoxifen then disrupts the Cre ERT‐HSP 90 interaction, leading to disassembly of the complex and releasing active Cre‐recombinase. Tumours induced in this experimental setup were most effectively induced when the Cre‐recombinase was induced during the early postnatal phase, but ineffectively when induced in adults. It was also more effective when the gene delivery was aimed at the subventricular zone (SVZ; a major neurogenic area in the CNS) than delivery to other brain regions [139]. A gene recombination directly within the stem/progenitor cell compartment of the SVZ [140] demonstrated also that the brain tumour phenotype was strongly influenced by the gene recombination, with gliomas arising from recombination of *Pten* and *p53*, and primitive, poorly differentiated embryonal tumours from a recombination of *Rb* and *p53*, or by recombining *Rb*, *Pten* and *p53*; however, these experiments also demonstrated the essential role of *p53* in CNS tumourigenesis, whereas the recombination of *Rb* and *Pten* alone did not generate CNS tumours [140]. Notably, a phenotype very similar to the ‘triple mutant’ gliomas [140, 141] with a primitive neuronal phenotype was later observed in human glioblastomas with a similar mutation profile [142], i.e. in this scenario the mouse model anticipated the discovery in human tumours.

In addition to the combination of mutations, also the cell of origin is a determinant of the brain tumour phenotype: a detailed study identified gliomas (in a *GFAP‐Cre‐Nf1; p53; Pten* model) originating from cells of the diencephalon and brainstem (i.e. ventral brain) to express Olig2 and PDGFRA, whilst the remaining tumours were more aggressive and showed a higher level of GFAP expression [137].

### 7.3 Medulloblastoma models

The initial medulloblastoma mouse models followed the discovery of frequent mutations of the patched (*PTCH*) gene (i.e. the sonic hedgehog (*SHH*) receptor) [143] and downstream signalling factors (*SUFU*, *SMO* and *GLI*) in medulloblastoma derived from the external granular layer [65, 66]. Prior to the generation of conditional knockout mice, these models relied on the inactivation of one *Ptch* allele [144, 145], stimulation of *Shh* signalling [146], or inactivation of *Gli* [147]. The first conditional knockout mouse model of medulloblastoma inactivated *p53* and *Rb* in the external granular layer of the cerebellum, taking advantage of the early, transient expression of GFAP in this cell population. *GFAP‐Cre*; *RB* ^*lox/lox*^; *p53* ^*lox/lox*^ mice developed medulloblastoma at a high penetrance, and provided direct proof of the developmental origin of medulloblastoma [54], and later studies showed that *Myc* amplification leads to a large cell anaplastic phenotype, striking parallel to human medulloblastoma [148, 149, 150]. At the same time, in addition to the *SHH/PTCH* pathway alterations, also the *WNT* signalling pathway was described in medulloblastomas [67]. This led to the concept that distinct signalling pathways and subpopulations determine the origin of medulloblastoma. External granular layer‐derived medulloblastomas are caused by alterations of the *SHH* pathway [151] whilst SVZ/GABAergic cell populations give rise to *WNT*‐type of medulloblastoma [152] followed by experimental proof in a mouse model [153].

## 8 Combining PDGFRA expression with Cre‐mediated inactivation of tumour suppressor genes

The discovery that stem/progenitor cells of the CNS (termed B‐type of SVZ, [154]) express platelet‐derived growth factor receptor alpha (PDGFRA), and respond to stimulation with PDGF with hyperplasia with features of gliomas [155, 156, 157], formed the basis of experimental approaches combining PDGF receptor stimulation (either by expressing the receptor or the ligand) in combination with Cre‐recombinase, in the context of tumour suppressor gene models, such as conditional *Pten* ^*lox/lox*^, *Rb* ^*lox/lox*^, or *p53* ^*lox/lox*^ mice. Delivering PDGF and Cre‐recombinase by retroviral or lentiviral vectors, these models developed rapidly growing high‐grade gliomas at high penetrance, making them highly suitable for analysis of glioma‐associated pathways such as Notch signalling [158], IDH mutation [159] or for the generation of versatile preclinical models [160, 161, 162].

## 9 Generation of CNS tumours with the transposon/transposase system

Like retroviruses, transposons are genetic elements that can mobilise within the genome. These mobile genetic elements harbour a ‘cargo sequence’, flanked by two inverted repeats/direct repeats. The cargo sequence contains a 5′ long terminal repeat of the murine stem cell virus, splice donor and splice acceptors, and polyadenylation sites on both orientations. The enzyme transposase can mobilise the transposon through ‘cut‐and‐paste’ mechanism, in which they are excised from one site in the genome and integrated into another site [163]. Retrotransposons transpose via an RNA intermediate and are classified into (a) LTR retrotransposons, which encode reverse transcriptase and transpose in a manner similar to retroviruses, and (b) non‐LTR retrotransposons, which are transcribed by host RNA polymerases and may or may not encode reverse transcriptase [164]. The main practical differences between transposable elements include cargo capacity, integration site preference, and the rate of ‘local hopping’. Cargo capacity varies greatly among transposable elements; this is an important factor to consider, particularly for delivery of complex genetic cargos or longer genes.

**Fig. 5:**
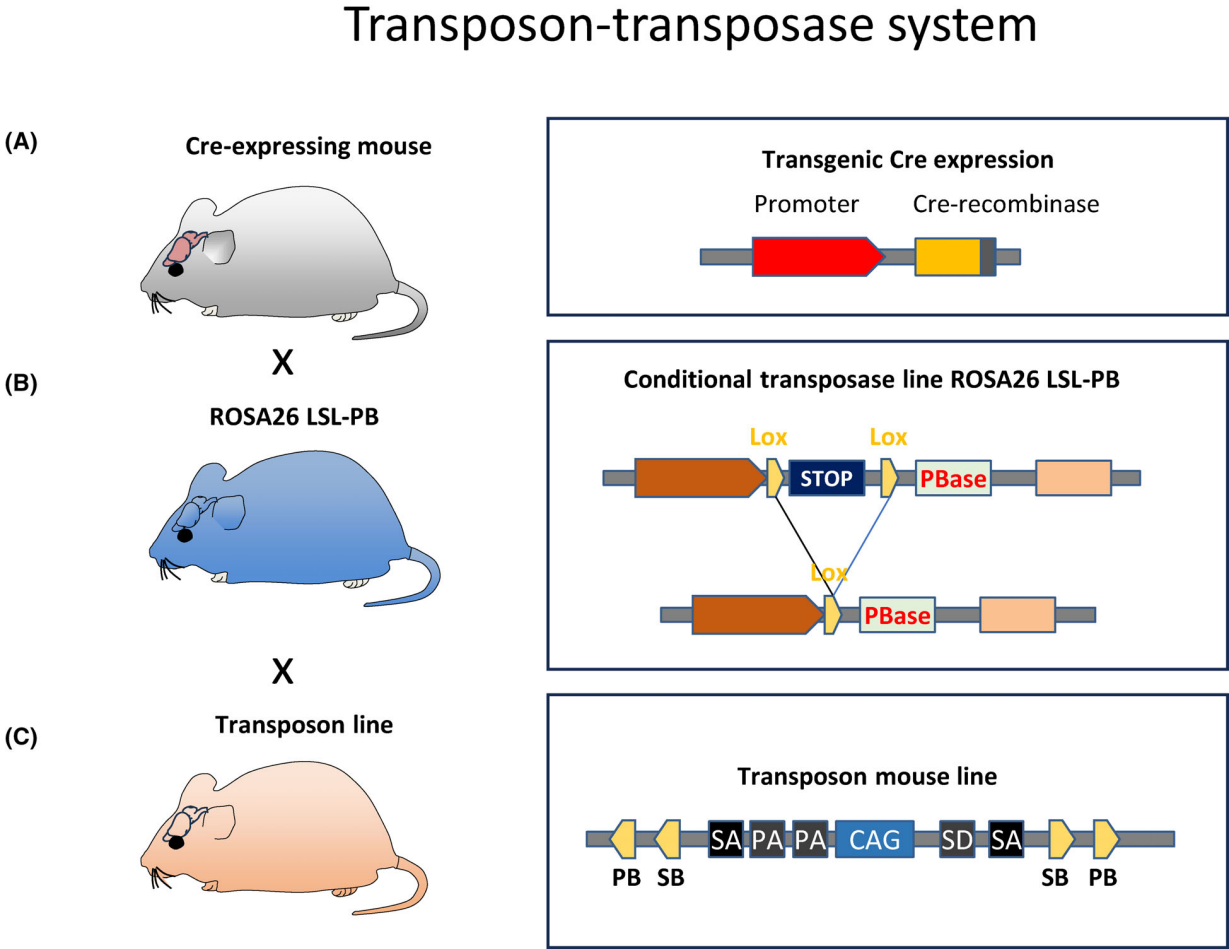
Brain tumour models, transposon‐transposase system (Sleeping Beauty, PiggyBAC). (A) Transgenic mouse lines expressing Cre‐recombinase are intercrossed with the Rosa26 lox‐stop‐lox conditional mouse (B), which is starts expressing piggyBAC transposases upon Cre‐mediated recombination. A third mouse line (C) expresses the transposons, which can be mobilised with the piggyBAC (PB) or the sleeping beauty (SB) transposases, and these can be engineered to cause gain or loss of function. SA, splice acceptor; SD, splice donor; PBase, piggyBac transposase; CAG, CAG promoter. PB, both piggyBac; SB, Sleeping Beauty (Adapted from [208]).

The sleeping beauty transposon (SB) system is used in insertional mutagenesis screens to identify known and novel oncogenes and cooperating cancer genes [165]. Mouse models require two components, one line expressing the transposon vector and the other line expressing the transposase enzyme (Fig. 5). When both are present in the same cell, the transposase recognises the inverted repeats of the transposon and excises it from the donor locus. The transposon can then insert itself at a TA dinucleotide region elsewhere in the genome. The transposase mouse line can be generated (for example) by ubiquitous expression within the *Rosa26* locus, or in a refined version, a Cre‐mediated conditional sleeping beauty system by targeting the *Rosa26* ^*lox/lox*^ site (Fig. 5A), to accomplish expression in a tissue‐specific manner. In comparison to retroviral insertional mutagenesis, the transposon shows a different insertional bias, and the tissue‐specific transposition enables cancer gene discovery across a broader range of tumours [166]. Using tissue‐specific or inducible Cre‐mediated approaches, especially in temporal control is possible, and using the sleeping beauty transposon as a tag, the mutated genes can be identified with high‐throughput PCR discovery methods [166]. The limitations are reduced efficiency with cargo sizes over 2 kb and variable expression across different target tissues. An alternative approach, piggyBac is more active, and the integration into the germline and into somatic cells is more random, it can carry larger cargoes (up to 9.1 kb), and inducible systems can achieve temporal and regional control of transposition [167].

SB mutagenesis screens have contributed to the characterisation of many cancers, such as gastrointestinal tract‐derived carcinomas [168], breast cancer [169, 170], hepatocellular carcinoma [171], peripheral nerve sheath tumours [172], osteosarcoma [173], melanoma [174], pancreatic cancer [175], or prostate cancer [175].

The induction of brain tumours was described in a mouse model with transgenic expression of a constitutively expressed SB (Rosa26‐SB 11) [176] and a *T2/ONC* mutagenic transposon [177], in the background of *p19Arf* ^+/−^; *p19Arf* ^−/−^ [178] or Blm (Bloom syndrome; genomic instability causing high cancer susceptibility) [179]. In these mice, high‐grade gliomas developed at high penetrance, and implicated CSF1 (colony‐stimulating factor) in tumourigenesis. In a similar setup, mice developed predominantly haematological malignancies and rare brain tumours, using *T2/ONC* low‐copy mice, mobilised by *Rosa26‐SB11* [180]. Both models however did not yield the targets commonly found in high‐grade gliomas, such as *PTEN*, *p53*, *ATRX*, *PDGFR*, or *EGFR*. A modified sleeping beauty mutagenesis was used by allografting genetically modified tumour cells, producing ‘mesenchymal’ subtype of glioblastoma; however, this model technically does not constitute a genetically engineered mouse. However, it identified multiple common insertion sites (CIS) that are also known in human CNS tumours, for example *MET*, *NF1*, or *PDGFRB* [181]. A conceptually different approach involving sleeping beauty used a sleeping beauty transposase together with an *Atrx* knockdown sequence, flanked by sleeping beauty recognition sequence. *Atrx* loss accelerated formation of high‐grade gliomas in the context of *p53* loss and *Nras* expression [182]. In humans, *ATRX* loss is frequently involved in formation of gliomas, in particular in combination with mutations in *IDH* (low and high‐grade astrocytoma, [183]), histone *H3 G34* (hemispheric high‐grade glioma) [184], *H3 K27M* (midline glioma) [184], or MAP kinase pathway activation (posterior fossa high‐grade astrocytoma with piloid features) [185]. The identification of cooperating oncogenes in medulloblastoma has been achieved in multiple studies. SB mutagenesis led to the discovery that medulloblastoma metastasis may have a divergent genetic makeup from the primary tumour, in that primary tumours may develop mutations after the formation of metastasis, pinpointing a critical barrier to targeted therapies which may therefore not be effective in metastatic seeds [186]. A mutagenesis screen in wild‐type and Trp53 R172H (an inducible lox‐stop‐lox allele with both dominant‐negative and gain‐of‐function properties [187]) mutant mice led to the discovery of multiple oncogenes involved in medulloblastoma progression, such as the transcription factor *FoxR2*, or *Tgif2* and *Alx4*, at the time newly identified putative oncogenes which are strongly expressed in the *SHH* subtype [188]. A medulloblastoma sleeping beauty transposon mutagenesis mouse model (*Ptch* ^+/−^, *SB100/SB68*, *T2Onc*) was also used to identify drug resistance candidate genes [189].

The additional utility of *PiggyBac* (PB) is a higher efficiency of transgene integration into target cells, and its larger cargo capacity. The H3 K27M midline glioma was modelled by in utero electroporation of the H3 K27M transgene into the stem/progenitor population in the subventricular zone (forebrain or hindbrain), alongside with CRISPR Cas9‐induced *p53* deletion. These mice developed diffuse gliomas in hindbrain or forebrain, depending on the gene delivery target area, but only when the transgenes were delivered in embryonic state. Instead, adult mice did not develop tumours, supporting the notion of the ‘window of opportunity’ for tumourigenesis, that is context‐dependent, i.e. differs between different driver mutations [190].

A realistic model of IDH‐mutant astrocytomas (characterised by *IDH*, *ATRX*, and *p53* mutations, and in grade 4 astrocytomas frequent additional deletion of *CDKN2A/B*) was accomplished by combining IDH (R132H) expression, and *Atrx* and *p53* inactivation using the sleeping beauty technology. This model confirms the clinical observation that the IDH mutation increases median survival in the absence of treatment, enhances DNA damage response (DDR) via epigenetic upregulation of the ataxia‐telangiectasia–mutated (ATM) signalling pathway, and elicits tumour radio‐resistance [191].

### 9.1 Conclusions and lessons learned from transposon/transposase models

The technical details with advantages and disadvantages of the different types of CNS tumour model systems, including their combination with other methods of genetic modification, such as *Cre‐lox* and CRISPR‐Cas9 have been discussed in detail elsewhere [192, 193]. In brief, the main advantages of the transposon system are (a) its genome wide coverage, (b) utility for loss of function and gain of function studies, both, in cell lines or primary cells, as well as in living organisms; (c) targeting of coding and non‐coding genomic regions, and (d) utility for selection of traits requiring multiple cooperating mutations. The disadvantages are (a) a potential bias regarding insertion site preference and a possible negative bias for genes which have their first ATG sequence in exon 1 or for genes of very small size, such as micro‐RNA; (b) not the full spectrum of mutations in human cancers such as point mutations and translocations are included and (c) the system can also create mutations due to re‐mobilisation [192]. In conclusion, transposon insertion mutagenesis has generated insight into, and understanding of, many dimensions of cancer development, progression including metastasis, and therapy response. This system has been used to model and understand many types of human cancers, however only limited numbers of CNS tumour models emanated from these studies.

## 10 CRISPR Cas9 technology

### 10.1 Introduction

The CRISPR (clustered regularly interspaced short palindromic repeats)‐Cas9 (CRISPR associated protein 9)‐system can introduce precise modification at specific DNA sites. When Cas9 introduces double‐strand breaks (DSB) the DNA repair machinery is activated. However, if these breaks remain unrepaired, there are two main outcomes, non‐homologous end joining (NHEJ) and homology‐directive repair (HDR) (or microhomology‐mediate end joining (MMEJ) repair [194]). NHEJ is the default repair pathway for DSB: when Cas9 cleaves both DNA strands, NHEJ stitches them back together, but often with errors. This causes small insertions or deletions at the cut site, causing disruption of gene function, and this can be harnessed for gene knockout studies. The other outcome, HDR, requires a repair template, usually a stretch of DNA with homology to the target site, and with this template being available, HDR can precisely repair the DSB. As a result, specific edits such as base substitutions or insertions, can be achieved, enabling gene correction, tagging or precise modifications [194, 195].

### 10.2 Models of CNS tumours combining CRISPR Cas9 technology with other genetically modified models

Whilst the growing level of sophistication of genome engineering technologies made it possible to manipulate nearly any candidate gene *in vivo*, the generation of genetically engineered mouse models usually involves relatively considerable efforts, including time‐consuming breeding schemes. The CRISPR–Cas system has further revolutionised cancer research by enabling very accurate, and at the same time highly flexible manipulation of the genome. Application of the CRISPR‐Cas system has been used to model cancers by inactivation of tumour suppressor genes [101, 102, 103, 196], engineering of indels [102] somatic point mutations [101, 104], and of complex genomic rearrangements, such as gene fusion events [105, 106]. The CRISPR Cas9 technology was used elegantly to determine how a genomic locus (rs55705857) that has been associated with increased risk to develop IDH mutant astrocytomas in humans (typically carrying IDH R132H mutations, *p53* mutations, and loss of function mutation of *ATRX*), can be modelled in an appropriate mouse model setting. To this end, and IDH‐mutant low‐grade glioma model was established using *Idh1 Lox‐Stop‐Lox (LSL)‐* ^*R132H*/+^ knock‐in mice [115, 197], and was crossed with a conditional *Trp53 LSL‐* ^*R270H*/+^ (p53 gain of function) mice. Finally, to enable CRISPR‐Cas9–mediated somatic mutagenesis of any other part of genetically relevant gene, these mice were crossed to *LSL‐Cas9‐GFP* mice, and Cre‐expressing lentiviral particles were delivered to the subventricular zone a newborn mice. Finally, the inactivation of *ATRX* was achieved by lentivirus‐*sgAtrx‐Cre* delivery, i.e. generating IDH (R132H), p53 (R270H), ATRX^−/−^ neural progenitor cells [198]. Of pathogenetic significance was the additional disruption of the genomic locus rs55705857, which increases penetrance and decreases latency of Idh1(R132H)‐driven glioma.

The combination of the CRISPR Cas9 technology with a library of sgRNA cloned into an AAV‐CRISPR vector enabled a high‐dimensional screening of sgRNA libraries [199], and identified functional suppressors in glioblastoma and a more comprehensive understanding of the genetic factors involved in glioma genesis. In this model, an AAV‐CRISPR vector encodes Cre‐recombinase under the control of the GFAP promoter, resulting in conditional expression of Cas9 and GFP in astrocytes (and other GFAP‐expressing cells, see above) after injection into a conditional Rosa26‐LSL‐Cas9‐GFP mouse. An sgRNA library targeting mouse homologues of top‐ranked pan‐cancer significantly mutated genes (SMGs) was then Cloned into the AAV‐CRISPR vector. The tumour induction efficacy was approximately 50%, and survival time approximately 130 days [199]. Next, targeted capture sequencing was used to map the mutational landscape and revealed diverse mutational profiles across different brain tumours, resulting in a comprehensive cataloguing of the genetic factors involved in gliomagenesis.

### 10.3 Combination of the CRISPR Cas9 approach with RCAS‐TVA

The RCAS‐TVA‐based approach (see chapter above and Fig. 2) delivers genes or shRNAs into a wide range of cell types, including CNS progenitors such as neural stem cells (nestin) astrocytes and GFAP‐expressing progenitors [116]. These two technologies were combined by intercrossing the *Rosa26‐LSL‐Cas9* knock‐in mice (*LSL‐Cas9*) [101] with NTVA or GTVA mouse lines. This mouse strain carries a floxed STOP cassette preventing constitutive expression of the downstream bicistronic sequences (*Cas9‐P2A‐EGFP*) and was generated to overcome the delivery challenges of the Cas9 enzyme to specific cell and tissue types. Crossing the *LSL‐Cas9* with the *NTVA* or *GTVA* transgenic mice generated *NTVA‐LSL‐Cas9* and *GTVA‐LSL‐Cas9* double transgenic mice [109]. This model now formed the basis for targeted gene knockdown using guide RNAs (sgRNA) to inactivate *p53*, *Cdkn2a*, and *Pten*, which are all involved in glioblastoma pathogenesis. The tumour penetrance was increased by adding an *RCAS‐PDGFB* vector. The ultimate goal however was the analysis of the effects of specific fusion proteins, *Bcan‐Ntrk1* (glioblastoma), *MYB‐QKI* (low‐grade glioma), but also *Braf* point mutations. This model was highly suitable for testing the effects of targeted drugs such as entrectinib (*NTRK* fusion) or trametinib (*BRAF* point mutations) [109].

### 10.4 Generation of CNS neoplasms by electroporation‐mediated gene transfer

The gene delivery into specific organs, or sometimes into specific regions, in a tightly temporally controlled manner can be a significant challenge, due to developmental regulation of gene expression and potentially lethal effects when expressed widely. *In utero* electroporation is a rapid technique to deliver transgenes into the developing brain [200, 201]. The disadvantages are a relatively complex procedure and potentially varied efficacy of gene transfer, and not always reproducible, consistent regional expression. This method has been successfully used to deliver CRISPR Cas9 constructs to the developing brain of genetically modified mice, resulting in the formation of medulloblastoma [103], H3.3 K27M paediatric high‐grade gliomas [202] or *Nf1*, *Pten* and *p53* mutant high‐grade gliomas [203]. Alternatively, a proof of concept study uses a combination of in utero electroporation with an adeno‐associated virus (AAV) mediated CRISPR Cas9 transfer [204].

### 10.5 Outlook and future directions

The utility of CRISPR Cas9 models has provided an unprecedented versatility of gene editing. Known limitations of current models however are reliance on error‐prone DNA repair. A recently developed technology, prime editing, provides an alternative, by encoding a Cre inducible prime editor in the mouse germline. The key advantages of prime editing are precision, versatility, and efficiency whilst having a broad range of applications. Prime editing allows for a highly precise and programmable editing of single nucleotide substitutions and indels, thus avoiding error‐prone DNA repair associated with CRISPR Cas9. Prime editors can also engineer a broader spectrum of mutations, and mediate all transition and transversion SNV, as well as defined indels whilst CRISPR Cas9 primarily induces DSB. Prime editing also yields a higher product purity and lower rates of unintended activity at off‐target loci than CRISPR Cas9. A recent model of lung and pancreatic cancer demonstrated the versatility by introducing multiple distinct variants of K‐ras or p53 [205, 206]. Whilst the above method focuses on the gene editing aspect of modelling, other important developments include the delivery to the CNS, to target regions or cell populations specifically and effectively. An adeno‐associated virus (AAV)‐mediated CRISPR screen was developed to investigate genetic factors regulating glioblastoma pathogenesis [199]. This technology and its potential refinement and sophistication will allow for delivery large libraries of target genes into the brain of conditional Cas9 mice, as shown in this study, and capture sequencing revealed mutational profiles that correlate well with patient data. At the same time, a general limitation of AAV, the effective crossing of the blood–brain barrier (BBB) has been addressed by the rational design of AAV9 variants displaying cell‐penetrating peptides (CPPs) on the viral capsid (AAV.CPP.16 and AAV.CPP.21) [207]. This significantly improved transduction efficiencies into the CNS, with up to 250‐fold increases in transduction across four mouse strains. One of the variants (AAV.CPP.16) enhanced transcytosis at the BBB and increased cellular transduction efficiency, making it more effective in delivering antitumor payloads in a mouse model of glioblastoma [207]. Undoubtedly, with continued and rapid development of gene editing technology, in combination with gene delivery methodology into rodent models, and on the other hand, powerful discovery pipelines on human cancers, will result in impactful combinations for modelling and understanding cancers, including those of the central nervous system.

## 11 Summary and conclusion

This article outlines the variety of model systems that have been used to generate brain tumours. Importantly, there is no ‘best solution’, or ‘ideal model system’. The model system can serve various purposes: (a) to establish a phenotype that resembles human CNS tumours. This was of particular relevance and importance in the early stages of cancer research, at a time when the molecular basis and the genetic makeup of human CNS tumours was only poorly understood; (b) to understand the histogenesis of human tumours, by introducing mutations into a presumed cell of origin. Some of these models recapitulated the histogenesis naturally, for example medulloblastoma arising from mice with a *Ptch* heterozygous genetic background. Others required an intentional combination of presumed cell of origin with a set of genetic lesions, for example the introduction of *Pten* and *p53* mutations into cells of the subventricular zone; (c) to recapitulate known human tumour types, such as IDH‐mutant astrocytoma. These proved particularly challenging, as many IDH‐mutant astrocytomas grow very slowly, and correspondingly, the most faithful models will take very long to develop such tumours. Therefore, the development of preclinical models for such low‐grade gliomas will inevitably be fraught with the conundrum of low growth rates and therefore make them difficult to fit in a preclinical setting requiring high throughput and short tumour latencies; (d) to develop preclinical models which have a high tumour incidence and a rapid growth, but have to compromise on the genetic makeup of the corresponding human tumour, for example by coexpression of growth factors such as PDGFB or introduction of deletions of tumour suppressor genes such as CDKN2A/B.

## Conflict of interest

The authors declare no conflict of interest.

## Author contributions

SB has conceived and written this review article.
